# Posture and gait in the early course of schizophrenia

**DOI:** 10.1371/journal.pone.0245661

**Published:** 2021-01-19

**Authors:** Valentina Presta, Francesca Paraboschi, Filippo Marsella, Valeria Lucarini, Daniela Galli, Prisco Mirandola, Antonio Banchini, Carlo Marchesi, Laura Galuppo, Marco Vitale, Matteo Tonna, Giuliana Gobbi

**Affiliations:** 1 Department of Medicine and Surgery–Human Anatomy, University of Parma, Parma, Italy; 2 Department of Medicine and Surgery, Human Anatomy, University of Parma, Parma, Italy; 3 Department of Mental Health, Local Health Service, Parma, Italy; 4 Department of Neuroscience, Psychiatric Unit, University of Parma, Parma, Italy; 5 AUSL Reggio Emilia, Reggio Emilia, Italy; 6 Movement Analysis Laboratory (LAM)–Parma University Hospital, Parma, Italy; Oslo Universitetssykehus, NORWAY

## Abstract

While correlations between postural stability deficits and schizophrenia are well documented, information on dynamic motor alterations in schizophrenia are still scarce, and no data on their onset are available yet. Therefore, the aim of this study was i) to measure gait pattern(s) in patients with schizophrenia; ii) to identify posture and gait alterations which could potentially be used as a predictive clinical tool of the onset of the disorder. Body composition, posture and gait parameters were assessed in a group of 30 patients with schizophrenia and compared to 25 healthy subjects. Sway area was significantly higher in the schizophrenia group compared to controls regardless of whether the participants were in eyes open or eyes closed condition. Gait cadence and speed were significantly lower in patients with schizophrenia, while stride length was similar. We concluded that the combination of an increased sway area (independent from eye closure) and a gait cadence reduction—in the presence of normal gait speed and stride length—might be considered peculiar postural and gait profile characteristic of early schizophrenia.

## Introduction

Motor dysfunctions have been associated to schizophrenia spectrum since the early characterization of the disorder. With the advent of the first medical treatments, motor abnormalities were primarily classified as side effects of antipsychotics. Consequently, even if odd movements and postures were part of 16 of 18 distinct historical criteria for schizophrenia diagnosis, they were absent from all modern diagnostic criteria [1, 2]. However, in the last decades, motor abnormalities such as altered postural control, postural instability, gait, and balance deficits have emerged as a core features of schizophrenia [3, 4], regaining a central role in the pathophysiology of the disorder [5]. The current approach is to investigate the relationship between motor abnormalities and neuroleptics/antipsychotics use, to disentangle primary motor deficits from secondary drug-induced motor disorders [6–8]. Motor symptoms have been identified in individuals at high risk for schizophrenia, in naïve-drug patients, in first-episode schizophrenia individuals, as well as in schizotypal personality disorder [2–5]. Moreover, abnormal involuntary movements in childhood and adolescence are predictive of later onset of psychosis [9, 10]. Recently, it has been stated that treating the schizophrenia in the earlier stages of disease could positively impact the treatment outcomes [11], therefore the recognition of specific motor markers might represent a valid tool for the early detection of the disorder and a possible treatment target. It is difficult to gather gait abnormalities in patients with schizophrenia, because of a large variability over time or the presence of confounding factors (age, body mass index, etc.) that limit a full and clear understanding. For example, the association of body mass index (BMI) and postural stability was recently investigated, and some authors reported differences in BMI or fat percentage [12–14] between people with schizophrenia and healthy controls, that could be impact posture and gait parameters [15]. However, previous research on different psychiatric conditions, including schizophrenia, described some recurrent features like slower gait, shorter stride length and balance problems in these individuals [16]. It is still not clear when these deficits emerge, but it has been suggested that they could arise in the earlier stages of disease [17, 18]. Indeed, postural changes were found when comparing early- vs late-stage schizophrenia, with—surprisingly- a higher degree of severity in the earlier disease stages [18]. All together, these findings strongly propose the motor dimension as a strong endophenotypic candidate for schizophrenia [5].

Quantitative analysis of postural and gait parameters may represent a strategy to identify a specific motor pattern in patients with schizophrenia. The search for a specific motor profile could help to shed light on the neurodevelopmental processes underpinning schizophrenia spectrum. In this connection, specific deficits in postural sway in schizophrenia have been shown in different conditions (i.e., open/closed eyes, open/closed base, challenging sensory conditions), possibly due to an impaired integration system of visual and proprioceptive signals [8, 19, 20]. According to literature, people with schizophrenia show a greater sway area, thus a reduced postural control in quiet standing conditions, as compared to healthy subjects, and the increase of sway area is more evident with the removal of visual input [8, 19]. Putzhammer *et al*. [7, 21, 22] assessed gait disturbances during dynamic conditions (walking) in patients with schizophrenia who vary on antipsychotic treatment categories (i.e., drug-naïve and treated with typical and atypical antipsychotics) and compared to healthy subjects, describing a reduction in gait speed (distance covered in meters per second), while cadence (number of steps per minute) did not change. Studies aimed at investigating postural control in schizophrenia patients found an increased centre of pressure (COP) parameters, that is sway area and path length, compared to healthy people [20, 23]. This result could be the consequence of an altered integration of sensory information, correlated with the inability to adjust the centre of gravity to maintain balance and posture in people with schizophrenia [5, 8]. Cerebellar pathways are critical for postural control. Particularly, the lack of cerebellar control (predominantly vermal-fastigial) on motor and cognitive functions “may hamper the individual’s ability to smoothly and automatically maintain the homeostatic, context-dependent responses that govern behaviour” [24]. Interestingly, cerebellar abnormalities in functional activation, volume, white matter integrity, functional connectivity and other alterations have been repeatedly observed in schizophrenia spectrum [25] and might explain core symptoms such as behavioural and emotional dysmetria [26]. On these bases the present study aimed to (i) extensively investigate gait and posture parameters in schizophrenia patients and, in a secondary analysis, (ii) to look across patients to capture a possible motor profile for the early detection of the disorder.

## Materials and methods

### Participants

Patients with schizophrenia were recruited, after informed consent, among individuals affected by schizophrenia seeking treatment at the *Integrated Mental Healthcare and Pathological Dependencies* (DAISM-DP, Parma, Italy), from 2016 to 2018. The diagnosis of schizophrenia was validated using the psychosis module of the Structured Clinical Interview for DSM-IV axis I disorder, clinical version (SCID-I CV) [27–30]. Exclusion criteria included current mental disorder related to a general medical condition (according to Axis III definition in DSM-IV), cognitive impairment (Mini Mental State Examination score lower than 25), and substance or alcohol abuse or dependence. The Structured Clinical Interview for DSM-IV (SCID-I CV) was also adopted to exclude comorbid Axis I diagnoses and lifetime or current substance or alcohol abuse. To provide a valid consent for the participants for this study, patients were included only after the resolution of the acute phase of illness was reached, defined as the achievement of a clinical stabilization phase with initial symptom response and reduced psychotic symptoms severity [31]. The latter was described as a reduction in psychoticism dimension (hallucinations and delusions) to a low to mild symptom intensity level [32]. Patients were continuously monitored and informed about their therapeutic interventions and clinical conditions. Based on time from first hospitalization, the schizophrenia group (SG) was subdivided in three subgroups: ≤5 years (early-term disease, ETD), 6–14 years (middle-term disease, MTD), ≥15 years (long-term disease, LTD) from the first hospitalization. Healthy subjects recruited from the local community as volunteers composed the control group (CG). Exclusion criteria from the control group were: a previous or family history of mental disorders, cognitive impairment (Mini Mental State Examination score lower than 25), and past or current substance or alcohol abuse or dependence. All the data about participants are reported in Tables 1 and 2. The study was approved by the Local Institutional Ethics Committee of Area Vasta Emilia Nord (AVEN, Emilia-Romagna region, approved on 12/07/2016 n 24150). All participants provided a written informed consent after a complete and exhaustive description of the study and its aims.

**Table 1:** Demographics, anthropometrics, and body composition characteristics of patients with schizophrenia and healthy controls.

	Schizophrenia group (SG, n = 30)	Control group (CG, n = 25)	*p*-Value
**Age (years)**	37.6 ± 13.2	36.5 ± 12.4	n.s.
**Gender (male/female)**	21/9	13/12	n.s.
**Height (cm)**^**a**^	171.8 ± 10.7	171.8 ± 7.5	n.s.
**Weight (kg)**^**a**^	79.7 ± 18.7	66.5 ± 10.4	0.003
**BMI (kg/m**^**2**^**)**^**a**^	26.8 ± 5.0	22.0 ± 3.5	<0.001
**PBF (%)**^**a**^	27.8 ± 9.4	21.8 ± 6.5	0.009

Values are shown as mean ± standard deviation (SD). Anova and *t*-student tests, data were considered statistically significant if *p*-Value was less than o.o5.

^a^SG, n = 29. BMI = body mass index; PBF = percentage of body fat.

**Table 2:** Schizophrenia subgroups according to the time from first hospitalization.

	Schizophrenia subgroups			*p*-Value
	Early-Term Disease (ETD) ≤ 5 years (n = 12)	Middle-Term Disease (MTD) from 6 to 14 years (n = 9)	Long-Term Disease (LTD) ≥15 years (n = 9)	
**Time from first hospitalization (years)**	2.8 ± 1.8	10.8 ± 2.4	28.1 ± 11.7	<0.0001
**Age (years)**	26.8 ± 6.0	38.6 ± 11.0	51.0 ± 9.0	<0.0001
**Gender (male/female)**	10/2	6/3	5/4	n.s.
**Height (cm)**^**b**^	175.5 ± 9.9	170.1 ± 12.3	168.3 ± 9.4	n.s.
**Weight (kg)**^**b**^	85.4 ± 20,4	73.6 ± 17.9	77.9 ± 16.6	n.s.
**BMI (kg/m**^**2**^**)**^**b**^	27.5 ± 4,6	25.1 ± 3.9	27.7 ± 6.7	n.s.
**PBF (%)**^**b**^	27.4 ± 9.0	27.5 ± 6.2	28.9 ± 13.3	n.s.

Values are shown as mean ± standard deviation (SD). One-way ANOVA, data were considered statistically significant if *p*-Value was less than o.o5.

^b^LTD, n = 8. BMI = body mass index; PBF = percentage of body fat.

### Anthropometry and body composition

Body height (cm) was assessed barefoot using a mechanical portable stadiometer, whereas weight, Body Mass Index (BMI) and body composition was assessed through a non-invasive bioelectrical impedance analysis system (BIA, InBody 230) and analysed using appropriate software (Lookin’Body Health care System, Biospace Co., Milan, Italy) (Tables 1 and 2).

### Stabilometry and gait analysis

Motor pattern was assessed in patients with schizophrenia and control group in both static and dynamic conditions. Static condition was analysed by using a stabilometric platform (PODATA 2.0). Patients stood barefoot, arms at their sides and open legs on the platform in a comfortable manner. Analysis lasted 30 seconds (GPS–Global Postural System). Measurements were performed in the same order for all participants: firstly, with open eyes (OE), followed by closed eyes (CE). Static assessments were analysed using COP parameters, including path length (mm) and 90% confidence ellipse area (sway area, mm^2^). These parameters were automatically measured by the stabilometric platform and sent to the dedicated software as raw data, then used for comparisons and statistical analyses. The stabilometric platform records the individual fluctuations or swayings described as variations of COP parameters, path length and sway area. Path length represents the total length of the COP trajectory during upright standing, and the sway area is the area covered by the individual swayings. The analysis of COP parameters is representative of the individual postural control and balance, thus greater variations may point at postural instability [33]. The dynamic condition was investigated through gait analysis. A wireless inertial sensor (G-Sensor®, BTS Bioengineering s.p.a, Milan, Italy) was applied to participants with a S1 level attached belt. The gait analysis was performed on a 13-meter-long path and participants were asked to walk back and forth of the walkway at a self-selected natural speed. The dynamic condition was repeated three times to obtain the most natural walking condition from subjects. The final third trial was used in further analyses because it is assumed to represent the most natural walking condition. Walking performance data were sent to a PC by Bluetooth and evaluated by BTS G-STUDIO software (BTS Bioengineering G-Studio®, Milan, Italy). Gait cycle analysis parameters included gait speed (m/s), cadence (steps/min), stride length (m), and percentage of stride length (%height). These parameters were automatically recorded by the inertial sensor during the walking performance and sent to the dedicated software soon after the end of each test; the collected data were used to compare the gait parameters among groups and analysed for the statistical significance.

### Statistical analysis

Demographics and anthropometrics comparisons between patients with schizophrenia and healthy controls groups were examined using One-Way ANOVA and *t*-tests. Both static and dynamic conditions were assessed using independent and/or correlated samples (*t*-tests). The sample size calculation was based on Matsuura *et al*. [6] and assumed a two-sided test (α = 0.05), by which a minimum of 23 patients were necessary to detect statistical differences between the two groups. After first comparison, patients with schizophrenia were divided by time from the first hospitalization. Time from first hospitalization refers to the first patient admission to psychiatric services. Duration of untreated psychosis (DUP) has not been considered because of the unreliable anamnestic information prior to the first referral to psychiatric services.

We performed an additional analysis of static and dynamic parameters of three subgroups compared with healthy controls. Since the sample size of each subgroup did not reach the statistical power, we considered these analyses as exploratory. One-way ANOVA was used to compare the three schizophrenia subgroups with the controls group, whereas gait speed and cadence were analysed performing *t*-tests to compare each subgroup to healthy controls group. Data were considered statistically significant if *p*-Value was less than o.o5.

## Results

In order to eliminate the potential confounding effects of different psychoactive medication on the results, we converted all antipsychotic therapy in Chlorpromazine equivalents, and we found no correlations with alteration in stabilometric and dynamic parameters.

### Patients with schizophrenia showed reduced static and dynamic postural control

**Fig 1:**
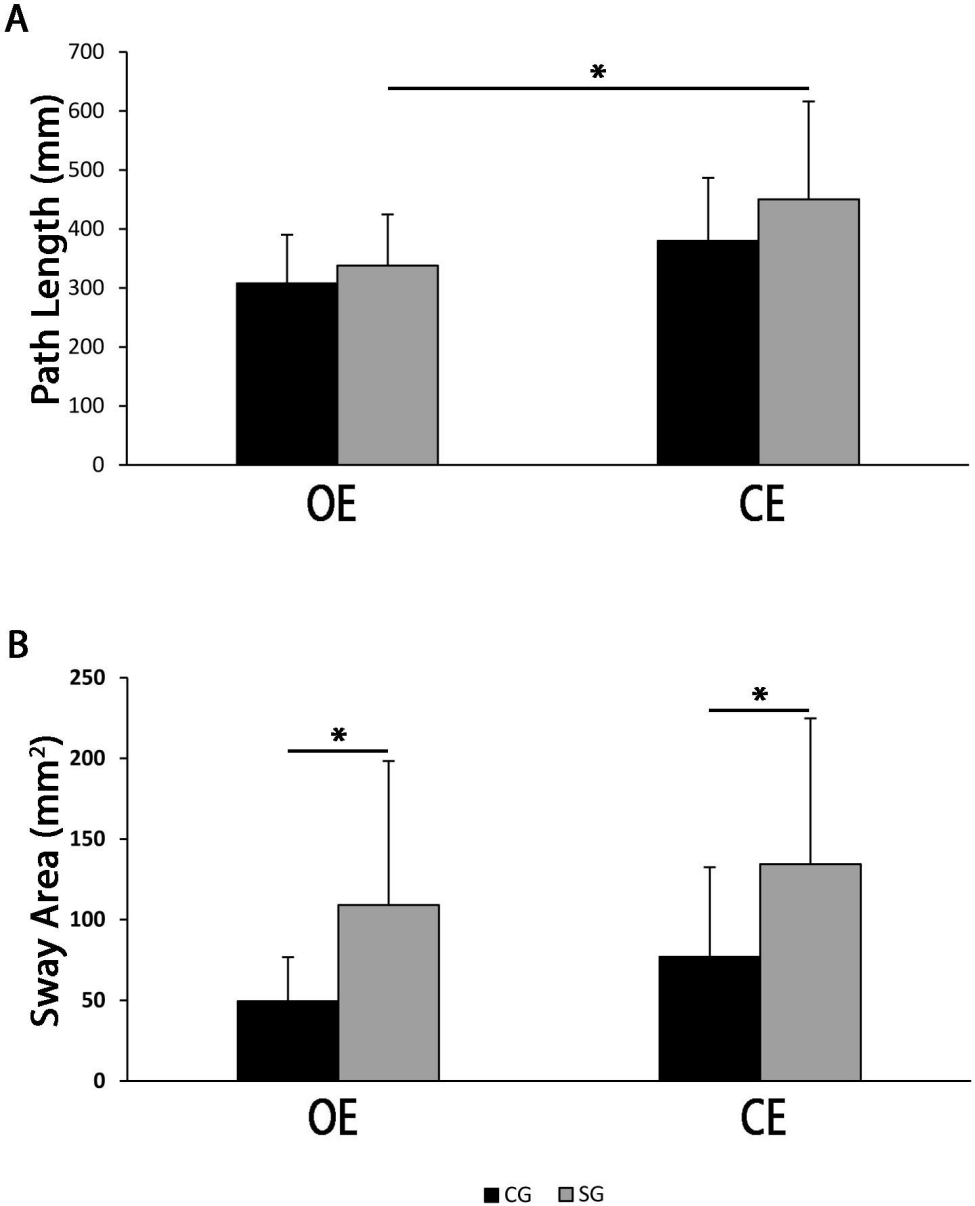
Path length (panel A) and sway area (panel B) for healthy controls (CG, n = 25) and schizophrenia group (SG, OE n = 27, CE n = 28). OE bars represent data for open eyes condition and CE bars represent the closed eyes condition. One-way ANOVA and Tukey HSD Test for post-hoc comparisons were used to compare path length and sway area between CG and SG in both open and closed eyes conditions. Data were considered statistically significant if *p*-Value was less than 0.05. *p<0.05.

**Fig 2:**
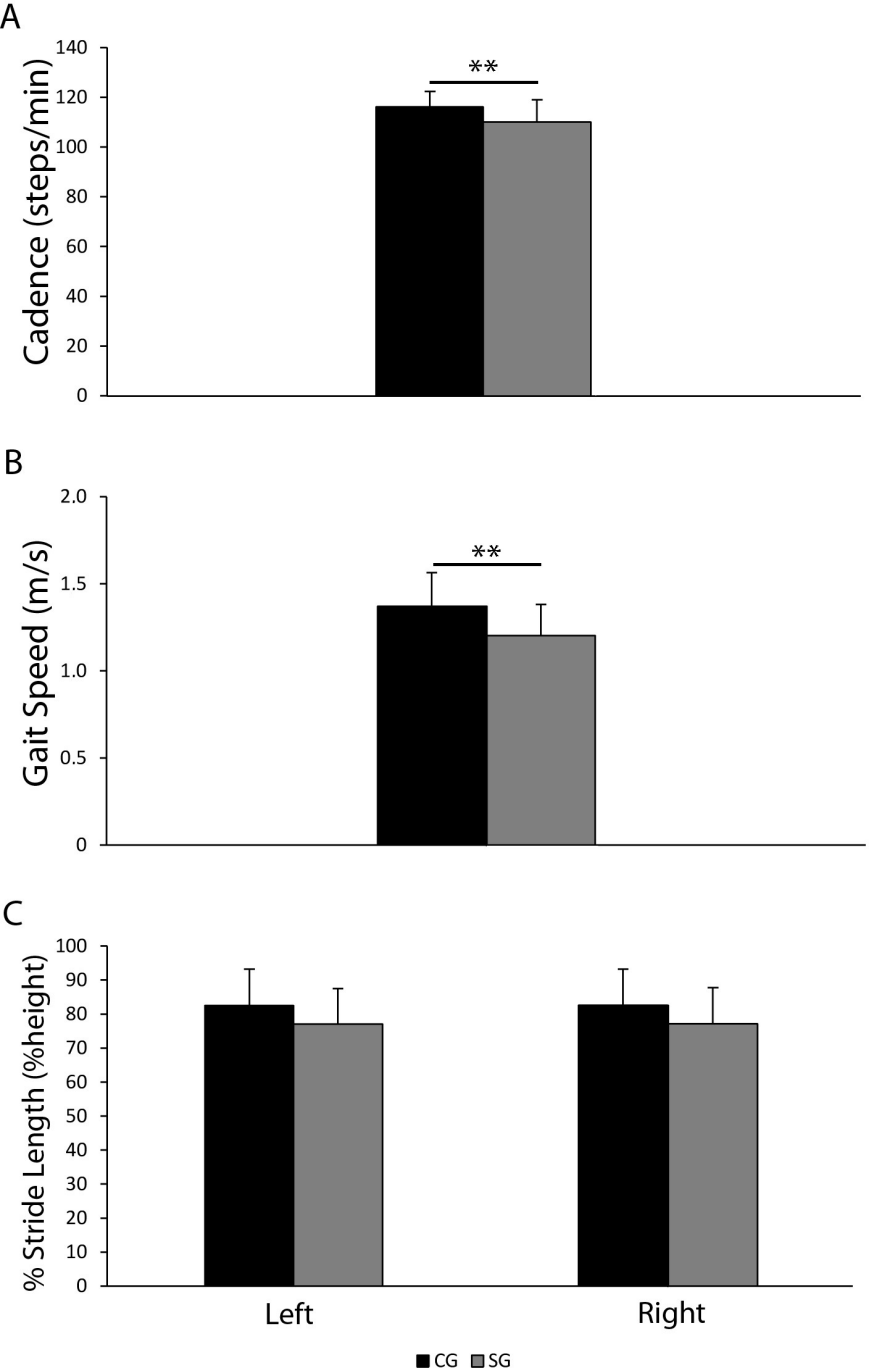
Cadence (panel A), gait speed (panel B) and percentage of stride length (% Stride Length, panel C) for healthy controls (CG, n = 24) and schizophrenia group (SG, n = 27). T-tests were performed between CG and SG for cadence and gait speed, one-way ANOVA for comparisons between left and right percentage of stride length between CG and SG. Data were considered statistically significant if *p*-Value was less than 0.05. **p<0.01.

Stabilometric parameters showed a significant increase in path length (Fig 1, panel 1A) from OE to CE condition only for patients with schizophrenia [SG, OE: M = 336.3, SD = 85.9; CE: M = 450, SD = 166.1, F(3, 101) = 7.49, p < .01], although within the same condition (OE or CE) patient path length was not statistically different as compared to controls (OE, SG: M = 336.3, SD = 85.9; CG: M = 307.8, SD = 82.6; CE, SG: M = 450, SD = 166.1; CG: M = 379.8, SD = 106.9). By contrast, sway area was significantly higher in the schizophrenia group both in OE and CE conditions as compared to healthy controls [OE, SG: M = 107, SD = 88.3; CG: M = 49.5, SD = 27.2; CE, SG: M = 134.4, SD = 90.4; CG: M = 77, SD = 55.6, F(3, 101) = 6.75, p < .05] (Fig 1, panel 1B). Gait cadence, speed, and percentage of stride length (dynamic parameters) were analysed as well (Fig 2). Patients with schizophrenia showed a lower gait cadence (SG, M = 110.1, SD = 8.5; CG, M = 116.2, SD = 6.2, t(49) = -2.81, p < .01) and speed (SG, M = 1.2, SD = 0.2; CG, M = 1.4, SD = 0.2, t(49) = 3.25, p < .01) (Fig 2, panels 2A, 2B) and a similar stride length (Fig 2, panel 2C), as compared to healthy controls, with no side preference. Of note, further ANCOVA analysis, aimed to test if BMI or age could be associated to the differences of gait speed between cohorts, confirmed its significant reduction in patients with schizophrenia.

### Sway area increased in the early stages of disease

**Fig 3:**
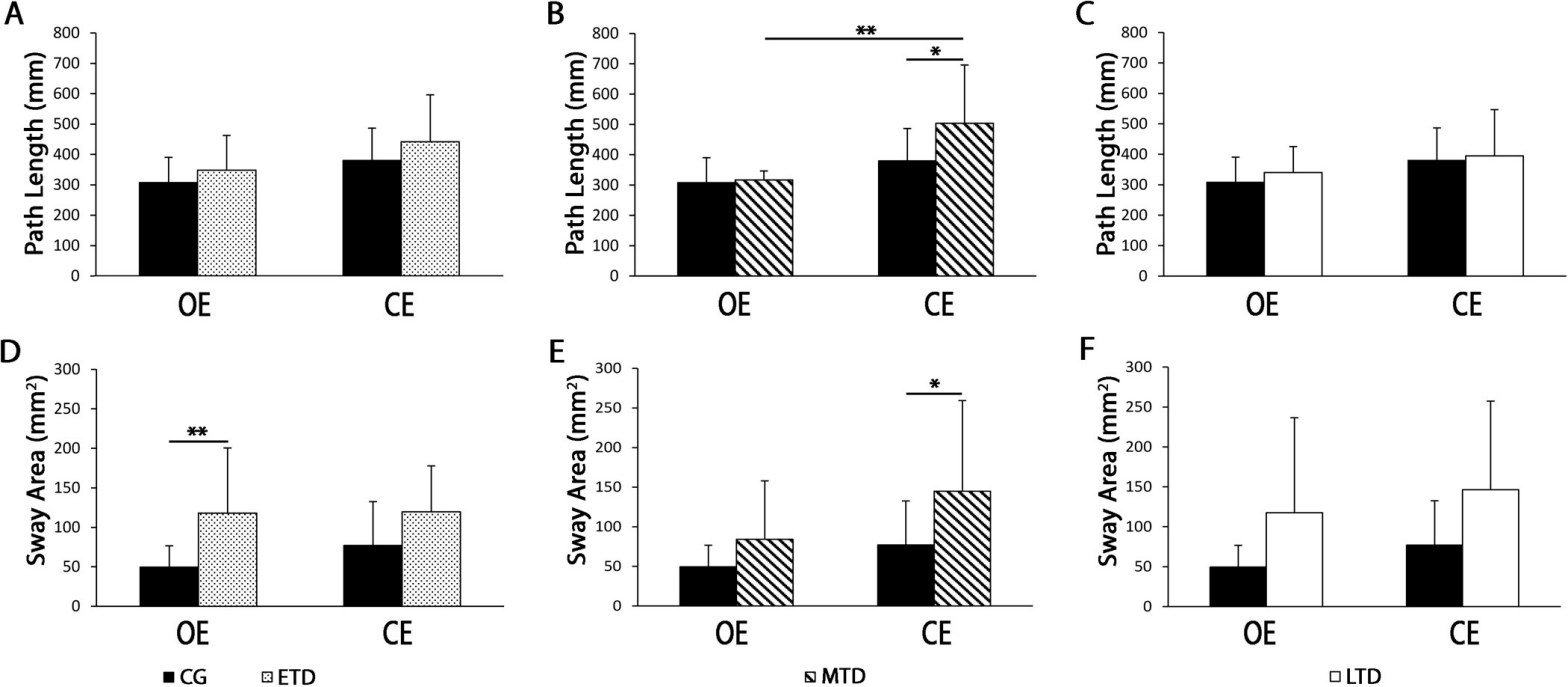
Path length (panels A-C) and sway area (panels D-F) for healthy controls (CG, n = 25) and schizophrenia group (SG) divided by time from first hospitalization (early-term disease, ETD, n = 12; middle-term disease, MTD, OE n = 8, CE n = 9; late-term disease, LTD, n = 7). OE bars represent data for open eyes condition and CE bars represent the closed eyes condition. One-way ANOVA and Tukey HSD Test for post-hoc comparisons were used for path length and sway area comparisons between CG and ETD, MTD, and LTD in both open and closed eyes conditions. Data were considered statistically significant if *p*-Value was less than 0.05. *p<0.05 **p<0.01.

Based on the time from first hospitalization, the SG was subdivided into three subgroups: ETD (≤5 years); MTD (6–14 years); LTD (≥15 years). With the limits of the small sample size subgroups, we decided to perform exploratory analyses to test the presence of early specific motor markers targeting schizophrenia progression. When each subgroup was compared to controls, a significant increase in path length emerged in the MTD group in CE condition (Fig 3, panel 3B) [CE, MTD: M = 503.7, SD = 192.6; CG: M = 379.8, SD = 106.9, F(3, 63) = 7.84, p < .05], that was not evident in the cumulative data analysis (see Fig 1, panel 1A), and from OE to CE condition (MTD, OE: M = 319.5, SD = 30.3; p < .01).

Sway area increase of the schizophrenia group was statistically different when comparing the ETD versus the healthy controls only in the OE condition [OE, ETD: M = 118, SD = 82.6; CG: M = 49.5, SD = 27.2, F(3, 70) = 6.81, p < .01]. Even all the data related to SG subgroups were higher than those of CG, a significant difference was found only in the MTD in the CE condition [CE, MTD: M = 144.8, SD = 114.5; CG: M = 77, SD = 55.6, F(3, 63) = 5.39, p < .05] (Fig 3, panels 3D-3F).

### Cadence and speed were the earliest gait parameters reduced in schizophrenia group

**Fig 4:**
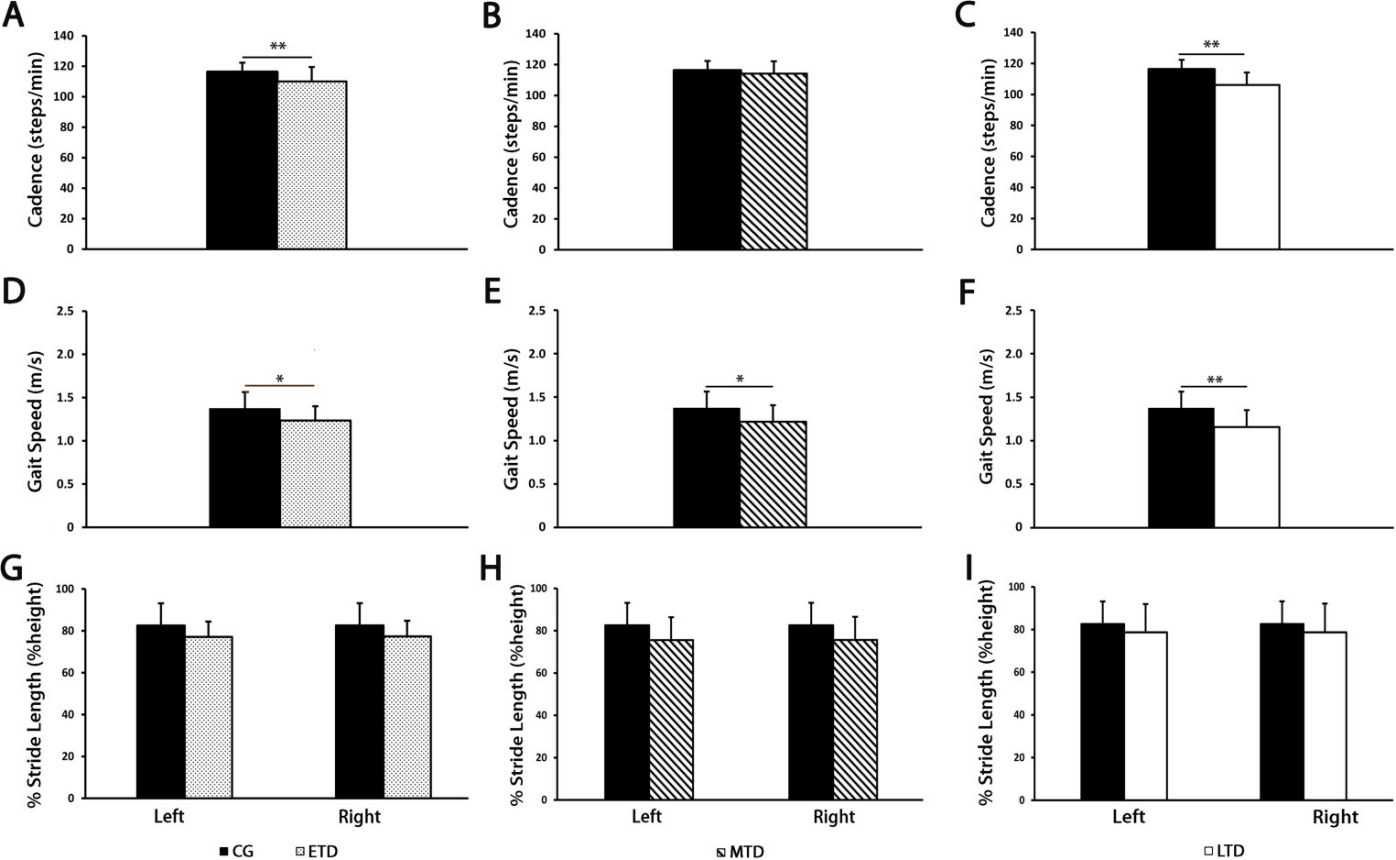
Cadence (panels A-C), gait speed (panels D-F) and percentage of stride length (% Stride Length, panels G-I) for healthy controls (CG, n = 24) and schizophrenia group (SG) divided by time from first hospitalization (early-term disease, ETD, n = 9; middle-term disease, MTD, n = 9; late-term disease, LTD, n = 9). T-tests were performed between CG and ETD, MTD, and LTD for cadence and gait speed; one-way ANOVA for comparisons between left and right percentage of stride length between CG and ETD, MTD, and LTD. Data were considered statistically significant if *p*-Value was less than 0.05. *p≤0.05, **p<0.01.

The analysis of dynamic parameters in the ETD, MTD and LTD subgroups are shown in Fig 4. Comparison with controls showed a reduction of gait cadence (Fig 4, panels 4A-4C) in patients with ETD and LTD [ETD: M = 110.1, SD = 8.3; LTD: M = 106.1, SD = 8; CG: M = 116.2, SD = 6.2; ETD vs CG t(34) = 2.49, p < .01; LTD vs CG t(31) = 3.85, p < .01], mirroring what observed in the cumulative analysis (cf. with Fig 2, panel 2A). Conversely, the reduction of gait speed observed in the whole population, was constantly found in all subgroups [ETD: M = 1.23, SD = 0.17; MTD: M = 1.22, SD = 0.19, LTD: M = 1.16, SD = 0.19; CG: M = 1.37, SD = 0.19; ETD vs CG t(31) = 1.89, p < .05; MTD vs CG t(31) = 2.07, p < .05; LTD vs CG t(31) = 2.85, p < .01], appearing in the early-term disease and becoming more evident in the late-term disease (Fig 4, panels 4D-4F). Stride length (Fig 4, panels 4G-4I) did not change in any patient subgroup.

## Discussion

Recent research in schizophrenia has been devoted to detecting early signs and symptoms of the disease [2, 34]. Even though motricity (posture and gait disturbances) appears as a promising candidate [3, 6–8, 19, 21–23, 35, 36], to our knowledge, an early motor profile has not been extensively investigated yet. In the present study, analysis of posture (static) showed that patients with schizophrenia had an increase in sway area which points to postural instability, as compared to healthy controls. This finding is consistent with previous studies [3, 5–8, 19]. However, in our study, patients did not increase sway when closing the eyes, (Fig 1, panel 1B), that is to say, the removal of visual input did not affect schizophrenia patients’ postural control. Similar findings were reported by Stensdotter *et al*. [37, 38] where patients with psychotic disorders had an increase in sway area compared to healthy subjects in the open eye condition, but this was comparable between healthy subjects and patients in the closed eye condition, suggesting a reduced ability to integrate the visual information for postural control. Since patients with schizophrenia show impaired integration mechanisms of vestibular, visual, and proprioceptive signals [6, 8, 20], our findings might be explained in the light of a decreased role of visual integration in the postural stability control.

With specific regards to dynamic parameters, we stratified patients into three groups (early, middle, and late) according to time from first hospitalization, to perform explorative analyses aimed to identify a postural/dynamic profile particular to the earliest phases of schizophrenia. We observed that middle-term disease participants did not change gait cadence in comparison to early-term and late-term disease. Our interpretation of these data is that the lack of statistical significance (see Fig 4, panel 4B) is mainly due to the limited sample size. Although not statistically significant, we can see a trend of cadence reduction in middle-term disease participants as compare to CG. Our findings show that cadence and speed are the earliest gait parameters reduced after the first hospitalization, whereas stride length does not change at all. Noteworthy, sway area of patients with schizophrenia showed a trend of constant increase as compared to healthy controls, which is independent from the time from first hospitalization; however, the underpowered subgroups showed significant increases of sway area only in the early and middle stages of disease (Fig 3, panels 3D-3F). Therefore, we hypothesize that increased sway area and gait cadence, together with speed reduction, may be primary signs of motor impairment in schizophrenia.

Brain imaging studies showed structural cerebellar dysfunctions in schizophrenia, responsible for impaired postural control and balance [4, 8, 19, 20, 39]. In fact, postural stability results from a complex integrating systems (visual input, vestibular and proprioceptive information) within cerebellar circuitries, working together to maintain both static and dynamic posture. We speculate that specific dysfunctions in cerebellar loops may be involved in deficits of postural control found in schizophrenia individuals. Results of this study might be limited by its cross-sectional design and by the small sample size. Moreover, SG and CG were not similar for body composition data (cf. Table 1), even if BMI did not affect our gait analyses. Therefore, larger longitudinal studies are needed to confirm our data.

Overall, we show that: i) sway area is increased, while gait cadence and speed are reduced in schizophrenia individuals; ii) the visual component of postural control is less relevant in patients with schizophrenia, as their balance–which is impaired–does not significantly suffer from eye closure; iii) increase of sway area and decrease of gait cadence and speed are the earliest detectable events in terms of motor alterations. These motor dysfunctions are not correlated with antipsychotic medications, and they can be considered as intrinsically linked with the psychotic disorder itself, as an endophenotype of the disease. Therefore, we speculate that they may be inherent to the neurodevelopmental impairment underlying schizophrenia spectrum which is a putative endophenotype of the disease.

In conclusion, the combination of an increased sway area independent from eye closure and a gait cadence as well as speed reduction in the presence of normal stride length might be considered as a motor profile specific to the early course of schizophrenia. The results of the present study highlight the importance of motor dimension as a core feature in schizophrenia. The recognition of specific motor markers might represent a valid tool for the early detection of the disorder and a possible treatment target. Future research is needed to investigate the clinical significance of motor profile in schizophrenia, particularly its possible association with specific symptom dimensions or syndrome configurations of the disease.

## Supporting information

S1 Table Path length and sway area in open eyes (OE) and closed eyes (CE) conditions of schizophrenia group (SG, OE n = 27, CE n = 28) and control group (CG, n = 25) during the stabilometric exam. Data are shown as Mean ± Standard Deviation.
(DOCX)

S2 Table Cadence, gait speed and left and right percentage of stride length of schizophrenia group (SG, n = 27) and control group (CG, n = 24) during the walking performance. Data are shown as Mean ± Standard Deviation.
(DOCX)

S3 Table Path length and sway area of schizophrenia subgroups (early-term disease, ETD, n = 12, middle-term disease, MTD, OE n = 8, CE n = 9, and late-term-disease, LTD, n = 7) and control group (CG, n = 25) in open eyes (OE) and closed eyes (CE) conditions during the stabilometric exam. Data are shown as Mean ± Standard Deviation.
(DOCX)

S4 Table Cadence, gait speed and left and right percentage of stride length of schizophrenia subgroups (early-term disease, ETD, n = 9; middle-term disease, MTD, n = 9; and late-term-disease, LTD, n = 9) and control group (CG, n = 24) during the walking performance. Data are shown as Mean ± Standard Deviation.
(DOCX)
