# Supplementary material for: Posture and gait in the early course of schizophrenia
Source: PLoS One. 2021 Jan 19;16(1):e0245661. doi: 10.1371/journal.pone.0245661 (PMC7815098; doi:10.1371/journal.pone.0245661)
Supplement: S1 Table — Data are shown as Mean ± Standard Deviation. (DOCX) [file pone.0245661.s001.docx]

**S1 Table.** Path length and sway area in open eyes (OE) and closed eyes (CE) conditions of schizophrenia group (SG, OE n=27, CE n=28) and control group (CG, n=25) during the stabilometric exam. Data are shown as Mean ± Standard Deviation.

|  | **SG** | | **CG** | |
| --- | --- | --- | --- | --- |
|  | **OE** | **CE** | **OE** | **CE** |
| **Path Length** | 336.3 ± 85.9 | 450 ± 166.1 | 307.8 ± 82.6 | 379.8 ± 106.9 |
| **Sway Area** | 107 ± 88.3 | 134.4 ± 90.4 | 49.5 ± 27.2 | 77 ± 55.6 |
