# Supplementary material for: Posture and gait in the early course of schizophrenia
Source: PLoS One. 2021 Jan 19;16(1):e0245661. doi: 10.1371/journal.pone.0245661 (PMC7815098; doi:10.1371/journal.pone.0245661)
Supplement: S2 Table — Data are shown as Mean ± Standard Deviation. (DOCX) [file pone.0245661.s002.docx]

**S2 Table.** Cadence, gait speed and left and right percentage of stride length of schizophrenia group (SG, n=27) and control group (CG, n=24) during the walking performance. Data are shown as Mean ± Standard Deviation.

|  | **SG** | **CG** |
| --- | --- | --- |
| **Cadence** | 110.1 ± 8.5 | 116.2 ± 6.2 |
| **Gait Speed** | 1.2 ± 0.2 | 1.4 ± 0.2 |
| **% Stride Length Left** | 77.1 ± 10.4 | 82.6 ± 10.6 |
| **% Stride Length Right** | 77.1 ± 10.6 | 82.6 ± 10.6 |
