# Supplementary material for: Posture and gait in the early course of schizophrenia
Source: PLoS One. 2021 Jan 19;16(1):e0245661. doi: 10.1371/journal.pone.0245661 (PMC7815098; doi:10.1371/journal.pone.0245661)
Supplement: S3 Table — Data are shown as Mean ± Standard Deviation. (DOCX) [file pone.0245661.s003.docx]

**S3 Table.** Path length and sway area of schizophrenia subgroups (early-term disease, ETD, n=12, middle-term disease, MTD, OE n=8, CE n=9, and late-term-disease, LTD, n=7) and control group (CG, n=25) in open eyes (OE) and closed eyes (CE) conditions during the stabilometric exam. Data are shown as Mean ± Standard Deviation.

|  | **ETD** | | **MTD** | | **LTD** | | **CG** | |
| --- | --- | --- | --- | --- | --- | --- | --- | --- |
|  | **OE** | **CE** | **OE** | **CE** | **OE** | **CE** | **OE** | **CE** |
| **Path Length** | 348.3± 114.3 | 442±  154.3 | 319.5±  30.3 | 503.7±  192.6 | 340.0±  85.1 | 394.9±  151.8 | 307.8± 82.6 | 379.8± 106.9 |
| **Sway Area** | 118± 82.6 | 119.7± 58.2 | 88.4±  77.7 | 144.8±  114.5 | 117.7±  118.9 | 146.3±  111.1 | 49.5± 27.2 | 77± 55.6 |
