# Supplementary material for: Posture and gait in the early course of schizophrenia
Source: PLoS One. 2021 Jan 19;16(1):e0245661. doi: 10.1371/journal.pone.0245661 (PMC7815098; doi:10.1371/journal.pone.0245661)
Supplement: S4 Table — Data are shown as Mean ± Standard Deviation. (DOCX) [file pone.0245661.s004.docx]

**S4 Table.** Cadence, gait speed and left and right percentage of stride length of schizophrenia subgroups (early-term disease, ETD, n=9; middle-term disease, MTD, n=9; and late-term-disease, LTD, n=9) and control group (CG, n=24) during the walking performance. Data are shown as Mean ± Standard Deviation.

|  | **ETD** | **MTD** | **LTD** | **CG** |
| --- | --- | --- | --- | --- |
| **Cadence** | 110.1 ± 8.3 | 114.2 ± 7.9 | 106.1 ± 8 | 116.2 ± 6.2 |
| **Gait Speed** | 1.23 ± 0.17 | 1.22 ± 0.19 | 1.16 ± 0.19 | 1.37 ± 0.19 |
| **% Stride Length Left** | 77.1 ± 7.3 | 75.6 ± 10.8 | 78.6 ± 13.3 | 82.6 ± 10.6 |
| **% Stride Length Right** | 77.3 ± 7.5 | 75.6 ± 10.9 | 78.7 ± 13.5 | 82.6 ± 10.6 |
